# The Prototype Monitoring System for Pollution Sensing and Online Visualization with the Use of a UAV and a WebRTC-Based Platform

**DOI:** 10.3390/s22041578

**Published:** 2022-02-17

**Authors:** Agnieszka Chodorek, Robert Ryszard Chodorek, Alexander Yastrebov

**Affiliations:** 1 Department of Applied Computer Science, Faculty of Electrical Engineering, Automatic Control and Computer Science, Kielce University of Technology, Al. 1000-lecia P.P. 7, 25-314 Kielce, Poland; a.chodorek@tu.kielce.pl (A.C.); a.jastriebow@tu.kielce.pl (A.Y.); 2 Institute of Telecommunications, Faculty of Computer Science, Electronics and Telecommunications, The AGH University of Science and Technology, Al. Mickiewicza 30, 30-059 Krakow, Poland

**Keywords:** field experiment, Internet of Things, unmanned aerial vehicle, pollution monitoring, urban environment, online visualization, WebRTC

## Abstract

Nowadays, we observe a great interest in air pollution, including exhaust fumes. This interest is manifested in both the development of technologies enabling the limiting of the emission of harmful gases and the development of measures to detect excessive emissions. The latter includes IoT systems, the spread of which has become possible thanks to the use of low-cost sensors. This paper presents the development and field testing of a prototype pollution monitoring system, allowing for both online and off-line analyses of environmental parameters. The system was built on a UAV and WebRTC-based platform, which was the subject of our previous paper. The platform was retrofitted with a set of low-cost environmental sensors, including a gas sensor able to measure the concentration of exhaust fumes. Data coming from sensors, video metadata captured from 4K camera, and spatiotemporal metadata are put in one situational context, which is transmitted to the ground. Data and metadata are received by the ground station, processed (if needed), and visualized on a dashboard retrieving situational context. Field studies carried out in a parking lot show that our system provides the monitoring operator with sufficient situational awareness to easily detect exhaust emissions online, and delivers enough information to enable easy detection during offline analyses as well.

## 1. Introduction

Nearly a hundred years ago, in 1923, the Journal of the American Medical Association reported a health risk from automobile exhaust gas in city streets [1]. During these hundred years, there has been both the development of techniques allowing for the reduction of the amount of emitted fumes and the harmfulness of exhaust gases, as well as a rapid increase in the number of vehicles being a source of the emissions. As a result, although a century has passed since this article [1] was published, car exhaust fumes are still a serious problem in our cities. However, due to general growth of ecological awareness, we are currently observing a great interest in air pollution, including exhaust fumes, which usually is manifested in the use of more ecological technologies and willingness to learn about the current level of pollution.

One of the methods of counteracting air pollution is to detect excessive emissions. Monitoring systems based on Internet of Things (IoT) devices and sensor networks have had a large share in this. The widespread use of these monitoring systems has become possible thanks to the use of low-cost sensors.

### 1.1. Pollutants

There are two main categories of air pollutants: gaseous ones and particulate ones [2,3]. Gaseous air pollutants include sulfur dioxide (SO${}_{2}$), oxides of nitrogen (NO${}_{x}$), and carbon monoxide (CO). From the group of oxides of nitrogen, nitrogen dioxide (NO${}_{2}$) dominates, the source of which is the combustion of fuels (certain coals and oil) and agriculture [4]. Particulate matter (PM) is defined as “a complex mixture with components having diverse chemical and physical characteristics” [2]. The PM is divided into three major fractions: particles less than 10 μm (PM10), particles less than 2.5 μm (PM2.5), and particles less than 0.1 μm (PM0.1). Particulate air pollutants are dust, soot, and smoke. Sources of PM are, e.g., residual oil and diesel fuel combustion, two-stroke vehicles [2]. The primary source of this air pollution is road transportation [5].

Harmful gases (e.g., SO${}_{2}$, CO, NO${}_{x}$) have an undesirable impact on the environment and, as a result, affect human health [6,7], especially in terms of heart and lung diseases. CO results in tissue hypoxia, hypoxic cardiac dysfunction, and death in high concentrations of CO. NO${}_{x}$ results in nausea, headache, asthma, and pneumonia. SO${}_{2}$ results in neurological damage, bronchitis, bronchial asthma, and emphysema [7]. It is worth noting that air pollution (both gaseous air pollutant, such as NO${}_{2}$ and CO${}_{2}$, and particulate air pollutants, e.g., PM2.5) raises the susceptibility to COVID-19 [8]. The details about the effect of short- and long-term exposures to PM2.5, PM10, O${}_{3}$, and NO${}_{2}$ are reported in the WHO’s Recommendations [9].

The WHO Air Quality Guidelines (AQG) identify the levels of air quality necessary to protect public health worldwide [10] and provides recommendations on air quality guideline levels for PM2.5, PM10, SO${}_{2}$, CO, NO${}_{x}$, and others. This document introduces AQG levels for the concentration of a pollutant in the air. Adverse health effects are assumed absent or minimal below the AQG level. AQG levels are associated with Interim targets (IT), understood as interim stages, when one strives to achieve the AQG level. AQG levels and ITs are determined for both long-term exposure (annual) and short-term (24-h) exposure. For example, in the case of NO${}_{2}$ short-term exposure (24-h), the document defines an AQG level of 25 μg/m${}^{3}$, and two ITI levels: IT1 of 120 μg/m${}^{3}$ and IT2 of 50 μg/m${}^{3}$.

The gaseous air pollutant most often measured by monitoring systems based on IoT devices is carbon dioxide (CO${}_{2}$) [11,12,13,14,15,16,17]. Often measured are also oxides of nitrogen [13,18,19] and volatile organic compounds (VOCs) [11,20]. Some IoT systems measure general air quality parameters, expressed by the Indoor Air Quality (IAQ) indicator [11] or the Air Quality Index (AQI) [21]. For measurements of the concentration of gaseous air pollutants, low-cost sensors often are used [22], such as the Waveshare MQ-135 [12,20,23] and other sensors of the Waveshare MQ family [23,24], the Bosch BME680 [21,24], the Bosch BME688 [11], and the Sensirion SGP30 [11]. In the case of particulate matter, the PM2.5 is usually measured [18,19,21,25,26,27], although some systems also measure PM10 [18,19,27,28] and PM1 [27]. For particulate matter measurements, such low-cost sensors as the Alphasense OPC-N2 [27], the Alphasense OPC-N3 [21], and the PMS7003 [28] are used. Some solutions also use expensive, customized sensors, such as the Dylos DC1700 [25,26].

Pollution measurements are usually accompanied by weather ones, together creating complete environmental monitoring. The most often measured weather factors are ambient temperature and relative humidity. Sensors used for these measurements are the Bosch BME680 [21,24], the Bosch BME688 [11], the Adafruit DHT11 [12], the Sensirion SHT11 [20,23], and the SparkFun RHT03 [17]. If the Bosch BME680 and the Bosch BME688 are used, atmospheric pressure also can be measured [11,21,24].

### 1.2. UAV and WebRTC-Based Platform for Monitoring Urban and Industrial Areas

The unmanned aerial vehicle (UAV) and Web Real-Time Communications (WebRTC)-based platform for monitoring urban and industrial areas, reported in our previous paper [29], combines IoT-based measurements with video from 4K camera and WebRTC [30,31,32], which was fully standardized this year [31,33] using the technology of native real-time transmission between Web browsers. One of the proposed new use cases of the WebRTC is the IoT [34] and, more precisely, the Web of Things (WoT), i.e., the integration of smart things with the Web [35].

The framework consists of an air station and a ground one. The air station includes the Raspberry Pi 4 Model B single-board computer (SBC), to which sensors are connected, and on which the WebRTC-based monitoring application is run. The monitoring application includes three services: the sensor service (customized according to the needs of the sensors used), the WebRTC video service, and the positioning service. This application is also an IoT hub, working as a server of the Message Queuing Telemetry Transport (MQ Telemetry Transport or MQTT) protocol. The full-stack WebRTC communication uses the Stream Control Transmission Protocol (SCTP) for near-real-time transmission of data from sensors and their spatiotemporal metadata, and the Real-time Transport Protocol (RTP) for real-time transmission of video data (the visual metadata for data from sensors). The mobility of the IoT is assured by a UAV, customized to perform the tasks of an IoT carrier in urban areas.

Data and spatiotemporal metadata are sent by the air station in MQTT messages through the WebRTC Data Channel; then, they are received by the ground station. The tasks of receiving, processing (if needed), visualization, storing (if needed), and retransmission to subscribers (if they exist) of data coming from the air station are performed by the WebRTC Multimedia and Monitoring Station (WMMS), which is a part of the ground station. The other part of the ground station, the Command and Control Console (CCC), is used for piloting the IoT carrier. The WMMS and IoT on board the air station communicate through a production network, which is the IEEE 802.11 wireless local area network (WLAN). The CCC and the IoT carrier communicates through the management and control network.

### 1.3. Main Contributions and Organization of This Paper

The experience gained during the design and implementation of the early prototype of the WebRTC-based flying monitoring system [36] was used during the construction of the UAV and WebRTC-based framework [37]. This framework was the basis of the mobile weather monitoring system [29]. This paper is focused on another purpose for the framework, which is the Kestrel, i.e., the system intended for widely understood environmental measurements carried out by air.

The main contributions of this paper are as follows:

- The building of a mobile weather monitoring system based on the UAV and the WebRTC-based universal platform, intended for pollution measurements accompanying the weather ones and including visual observations of the environment;
- The writing of visualization software able to take full advantage of contextual communication offered by the WebRTC and to put received IoT data, video, and spatiotemporal metadata into a common situational context;
- Field experiments aimed at conducting functional tests of the system in conditions of bad and good air quality.

The rest of this paper is as follows. Section 2 presents the air station, Section 3 describes the MQTT topics defined for the Kestrel, and Section 4 is devoted to the online visualization. Section 5 discusses the flight plan. Section 6 describes functional tests while Section 7 presents examples of retrospective analyses of collected data. Section 8 shows the Kestrel against the background of the other solutions known from the literature, and Section 9 summarizes this paper.

## 2. Kestrel

Kestrel was built using the UAV and WebRTC-based universal platform, presented in this paper [37], retrofitted with a set of environmental sensors. This section presents the main features of the Kestrel, such as applicability, contextual communications, and sensors retrofitting onto the base platform.

### 2.1. Applicability: Nomen Est Omen

Kestrel is a flying monitoring system intended for both measuring environmental parameters such as weather and air pollution, and for the visual observation of the surroundings in urban and industrial environments. The main areas for the application of the proposed system are the online environmental monitoring of current weather and current pollution, especially in places where there are no professional stationary measurement stations, filling the gap between these stations and systems based on voluntarily-carried devices or crowdsensing.

The Kestrel detects cars with excessive emission in two stages. First, it selects an area where the excessive emission occurred and then selects cars that could be a source of this emission. Often, the car, which is a source of emission, can be identified visually due to white or black smoke from the exhaust pipe, especially when starting a cold engine. Since the flying monitoring system “hunts” for sources of abnormal air pollution, it was named after the common kestrel (Falco tinnunculus)—a bird of prey that is able to adapt to the urban environment and increasingly inhabits urban areas, where it hunts pigeons and rodents. The system enables both online monitoring of the air quality of a patrolled area and, thus, the online detection of excessive emissions, and the storing of monitoring data for further processing and visualization, which supports offline detection and retrospective analysis.

**Figure 1:**
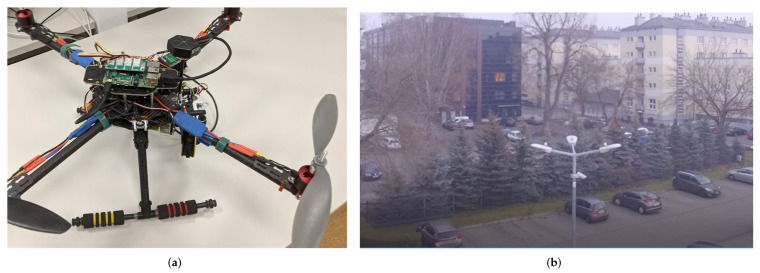
The Kestrel: (**a**) the air station of the UAV and WebRTC-based universal framework standing on the laboratory table; (**b**) the Kestrel when working in its “natural environment” (a picture captured from the Manta MM9359FS camera).

Figure 1 shows the Kestrel’s air station (the air station of the UAV and WebRTC-based universal framework, which was complemented with the set of sensors) and the picture made by the Manta MM9359FS camera, captured in late autumn when the air station was flying over the parking lot in light fog.

### 2.2. Contextual Communications

Contextual communications are such transmissions of data coming to the communication hub that enable the preservation of a situational context. The enabler of the contextual communication is the convergence of the IoT and real-time communication [38], able to put associated data from sensors and metadata (in Kestrel: spatial, temporal, and visual) in a common situational context. In the case of the proposed pollution monitoring system, contextual communication was achieved with the use of the full-stack WebRTC application, which enables transmission of an aggregated stream that consists of a real-time video stream (captured from the 4K UAV camera) and a non-real-time flow of data (coming from sensors and positioning module) in a shared channel.

Each sensor datum, each spatial datum, and each video frame are associated with a timestamp. In the case of a sensor datum, the timestamp is the time a given datum is sent to the communication hub, read from the system clock. In the case of a video frame, it is the time of the creation of the first byte of this frame, read from the video card clock.

Note that the temporal metadata used by the sensor service, and the temporal metadata used by the WebRTC video service, differ both in meaning and origin. A time metadatum that accompanies a sensor datum comes from the system clock and denotes a time of gathering of this sensor datum by the SBC, while a time metadatum that accompanies a video frame comes from the video card and denotes the time of creation of the first byte of this frame. Precise synchronization of real-time video stream and near-real-time data flow needs prior synchronization of these two clocks.

Each sensor datum and video frame are time-correlated with an approximate position of data gathering. Considering that the accuracy of the positioning module is up to 0.5 m, there is no necessity to use exact position.

The full situational context offered by the Kestrel includes exhaust gas concentration associated with position, particular matter information, video information, and weather conditions. This context is transmitted to the ground using WebRTC technology and then retrieved on the dashboard of the WMMS part of the ground station. Due to the limited space on the dashboard, only the spatiotemporal context of the exhaust gas concentration and the visual one need to be displayed.

### 2.3. Sensors

Sensors should be attached to the UAV and WebRTC-based platform according to the goals of any particular mission. The platform is able to work with many sensors, connected to the SBC via the analog input of the SBC, the Inter-Integrated Circuit (I${}^{2}$C) bus, or the serial Universal Asynchronous Receiver/Transmitter (UART) interface.

In the case of low-cost gas sensors, Kestrel can work with a whole family of popular analog solid state sensors. They are as follows: the propane–butane and hydrogen MQ-2 sensor; the alcohol detector MQ-3; the methane sensor MQ-4; the propane-butane sensor MQ-5; the LPG, isobutane, and propane sensor MQ-6; the carbon monoxide sensor MQ-7; the hydrogen sensor MQ-8; the carbon monoxide, alcohol, and gasoline sensor MQ-9; and the alcohol, benzene, and ammonia sensor MQ-135. The Kestrel is equipped with both a software interface (a part of the monitoring software) and a hardware interface (socket 1 × 4 pin, raster 2.54 mm) adapted to work with these types of sensors. The socket is powered with a voltage of 5 V, which is required by the heaters of the gas sensors. The power supply must also render a minimum 200 mA of current, which is a relatively large value when comparing it with, for example, temperature and humidity sensors.

The Kestrel is equipped with a set of sensors suitable for the measurements it has to perform during a given mission. If the mission is to detect combustion gases generated by cars in a parking lot, the base Kestrel’s gas sensor, namely, the MQ-135 solid state one, is used. When it is necessary to check, for example, gas leaks, the MQ-2, MQ-4, MQ-5, or MQ-6 sensor is used depending on the type of escaping gas.

Other sensors can also be connected to the I${}^{2}$C and UART interfaces. Typically, dust/air pollution sensors, such as the HM3301 or PMS7003 are connected via the I${}^{2}$C and UART interfaces, respectively. Weather sensors, which are also used in the Kestrel, are connected through the I${}^{2}$C interface.

Table 1 shows input devices used during the field experiment (functional tests) described in this paper. The Kestrel is equipped with a minimum set of sensors that include the Waveshare MQ-135 gas sensor, which is considered to be the basic Kestrel’s sensor. The MQ-135 is used for the detection of excess concentrations of exhaust gases. Some combustion products, especially of Diesel engine, are seen in PM2.5 monitoring; so, the Kestrel was equipped with the Seeed Studio HM3301 sensor, which measures particulate matter. The Measurement HTU21D temperature and humidity sensor was used for assessing current environmental conditions. Data from the HTU21D are used for improving the accuracy and stability of the measurement from the MQ-135 sensor in different temperature and humidity conditions [39].

### 2.4. Location and Calibration of the Gas Sensor

The measurement of pollutants with the use of UAV-based systems leads to many opportunities and many challenges [17,40,41]. One of the challenges is what influence the airflows produced by the rotors have on sensor measurements [17,40,41]. The presence of strong airflows requires proper selection of the place where the sensors should be mounted [17,40].

The optimal location of the sensors requires a study of the airflows produced by the rotors and the analysis of the quad-rotor aerodynamics [17,41] and the hexa-rotor aerodynamics [40]. Such studies, performed using simulations [17,42,43,44,45,46] and experiments in the real environment [17,40,41,45,46,47,48,49], showed that the calmest place, with the weakest airflow, is the middle part of the UAV. This applies to both small, several-hundred-gram UAVs [17], to medium-sized ones [41,42,45,47,48] and large, several-kilogram ones [40] as well.

The authors of this paper [17] determined the optimal location of the three environmental sensors: the luminosity one, the temperature and humidity one, and the CO${}_{2}$ gas sensor. This determination was carried out on the basis of the results of a wide spectrum of experiments: from simulation ones, through laboratory tests, to field tests in an isolated, controlled environment (greenhouse). The result of these works was the designation of areas suitable for the placement of specific sensors. We used these experiences while building the Kestrel. In particular, the pollutant sensors were placed in the center of the UAV, as was recommended in this paper [17].

The results of the experiments with a stationary drone, during which the propellers of the drone were turned on and off, showed that this placement of the sensors can collect the environmental variables at a satisfactory level [17]. The measurement error never exceeded 4% [17], which from Kestrel’s point of view is perfectly enough.

The MQ-135 sensor, which is the basic Kestrel sensor used for the detection of excessive emissions of exhaust gases, is factory precalibrated and no additional calibration is needed for this purpose. The sensor is operated in accordance with the terms of use. It is necessary to calibrate the sensor for each gas type [50]. This is usually performed using formulas taken from the literature. During experiments described in this paper, we used formulas taken from [50]. Before taking measurements, it is heated up for 24 h at the air station. It can be powered from two sides, and Kestrel’s air station enables dual powering of the MQ-135. However, measurements with the use of this type of sensor are sensitive to environmental conditions.

Typical for this gas sensor, temperature and humidity drift are taken into account by monitoring the applications that run on the air station. Data coming from the Measurement HTU21D temperature and humidity sensor are the basis for determining the correction for the measurements of the concentration of exhaust gases. Corrections are calculated according to the formula given in [39].

### 2.5. The Sword and Shield Problem

According to the concept of sword and shield, the development of systems to combat something (“swords”) is accompanied by a continuous development of defensive systems (“shields”), which in turn, entails a further development of “swords”. Therefore, it should be expected that the widespread detection of excessive emissions of exhaust gases based on semiconductor-type gas sensors will result in the emergence of systems counteracting such detection. As long as the cost of effective counteraction is high enough that it cannot be used on a mass scale, the use of “shields” will not be a statistically serious problem. However, if they are applied on a massive scale, the “sword” will have to be changed.

There are three potential directions for the development of “shields”, two of which we consider important—these are an attack on the pollution sensors and an attack on the IoT carrier. We assume that the third direction—an attack on the location system—is a much wider problem than the one discussed here, and appropriate solutions are already being prepared to counteract such attacks.

The attacks on gas sensors by, as an example, spraying with suitable chemicals or adding suitable additives to the fuel, may result in the need to replace the currently used sensors with sensors with better selectivity or the need to detect excessive emissions of exhaust gases on the basis of a fusion of information coming from a set of sensors. Kestrel supports such replacement through the use of the universal platform for fast prototyping [37] as the base for building this monitoring system. This gives the possibility of a quick and easy sensor change, also when a single sensor is replaced with a set of sensors. Moreover, the Artificial Intelligence (AI) hardware accelerator connected to the SBC, in which the Kestrel’s air station can be equipped [37], supports on-board data fusion, which can be used for removing deliberately introduced detection disturbances.

At present, however, the greater danger seems to be the attack on the carrier and the physical elimination of the air station by knocking it out of the sky. For this reason, Kestrel’s flights must be performed at a minimum altitude of 5 m, as required by the regulations and recommendations for UAV operations. This is the height where a human is unable to reach the UAV with his hand while jumping. In our opinion, typical patrol flights and sweeping of a patrolled area should be carried out at a higher altitude, so that a malicious person who throws something at the air station would not be able to throw it high enough.

In experiments described in this paper, Kestrel’s flights were carried out at the altitude of 5 m (in order to better identify and locate the emission source) and 15 m (sweeping the patrolled area). Note that the Kestrel is not intended for precise measurements. It was built to preventive patrolling of a given area and for determining the sources of excessive emission, which will then be verified by other entities with the possibility of precise measurements from the ground or air.

## 3. MQTT Topics

MQTT is the protocol of the application layer of the Open Systems Interconnection (OSI) model, originally intended for machine-to-machine communication [51,52]. This popular industry standard is often used in IoT systems because of its scalability and easy-to-implement, light-weight mechanisms. The MQTT works in the classic client–server model. In the case of the IoT, the client is an IoT device, and the server is the IoT broker. MQTT messages that convey the client’s data are sent using the MQTT publish method to registered subscribers on a given MQTT topic.

The MQTT topic is an 8-bit Unicode Transformation Format (UTF-8)-encoded unique text string identifying messages coming from a given device, placed in a given location. The MQTT topic typically has a hierarchical structure. The one created for the purposes of the Kestrel monitoring system identifies a measurement (e.g., the text string “temperature”) made by the device located on-board (“devonboard”) the IoT carrier (“UAV1”). The complete MQTT topic is made going down the hierarchy (here, “UAV1/devonboard/ temperature”). The use of an MQTT topic unequivocally identifies the source of a given datum and, also unequivocally, associates the flow of data coming from a given source with flows of their spatiotemporal metadata (Table 2).

A given datum and its spatiotemporal metadata are sent on the same MQTT topic. This means that although data and accompanying metadata come from different physical sources, they are identified by the same MQTT topic, which can be considered as a logical source; this causes the data and accompanying metadata to be identified by the same MQTT topic, which can be considered as the same logical source. To enable differentiation of physical sources within the MQTT topic, the next level of the hierarchy of source identifiers was introduced, i.e., the MQTT subtopic. The values of the subtopics text strings include the following:

- “value”, which denotes the value of a datum coming from a source identified by the MQTT topic;
- “time”, which denotes the approximate time of reading of this datum by the SBC;
- “position”, which denotes the approximate position of the air station during a gathering of this datum by the SBC.

As an example, if temperature measurements are sent between the air station and the ground one, the temperature read from the Measurement HTU21D sensor and the time of reading of the temperature by the SBC are identified by the same MQTT topic and two different subtopics (“value” and “time”). Then, the full text strings are as follows:

- “UAV1/devonboard/temperature/value” for temperature data;
- “UAV1/devonboard/temperature/time” for temporal metadata;
- “UAV1/devonboard/temperature/position” for spatial metadata.

The other situation can occur where the current position, given by the Waveshare SIM7000E positioning module, will be sent by the positioning service and by the sensor service. In the first case, the positioning metadatum will be labeled by the positioning topic, and in the second case, it will be labeled by the same topic as the accompanying datum (Table 2). In both cases, the positioning metadatum is identified by the “position” subtopic. Note that the sources of the current position identified by two different MQTT topics also are different. The current position datum comes from the positioning module, and the current position metadatum is read from the buffer.

## 4. Online Visualization

This section describes the layout of the dashboard displayed on the ground station and discusses the customization of the online visualization of the situational context.

### 4.1. Dashboard

**Figure 2:**
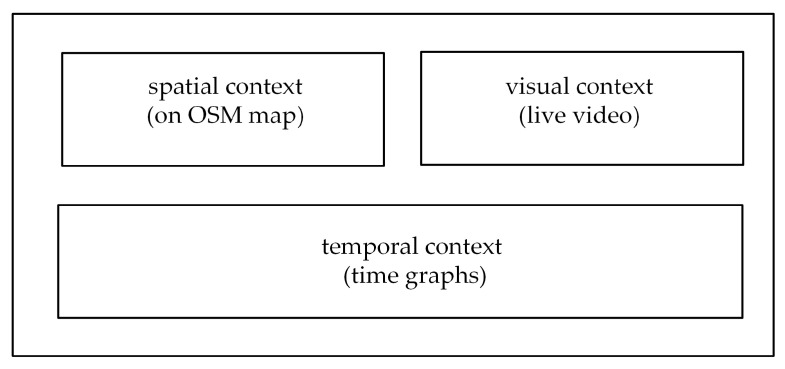
Layout of the dashboard.

Data are visualized on a dashboard, which is displayed on the monitor of the WMMS. The framework [37] introduced the division of the dashboard into three areas, each referring to a specific context (Figure 2). The two upper areas visualize a spatial context of received data and visual data. These areas are fixed-size windows, in which the current position of the UAV and the live video from a pan-and-tilt gimballed UAV camera are displayed. The position of the UAV is drawn on the map of the parking lot. The map is downloaded from the OpenStreetMap (OSM) repository, and the UAV is symbolized by an inverted triangle. Movements of the air station are visualized in the classic form of the static symbol of the vehicle (here, air station), which stays at the center of the fixed window, and movements of the underlying map. The processing of the OSM maps is made with the use of the Leaflet library. The current position of the UAV is delivered by the positioning service.

The video information, delivered by the WebRTC video service, is displayed in the upper-right, fixed-size window, and optionally also on an external monitor connected to the WMMS. It is worth remarking that although the WebRTC video service is a real-time one, compression and coding causes the displayed video context to be delayed in relation to the data processed and sent by the two other services. In the case of the Kestrel, video information is of auxiliary importance; so, there is no necessity to synchronize data for the purposes of online visualization. Such a need may arise during retrospective analysis.

The bottom area of the dashboard shown in Figure 2 was intended for the visualization of a temporal context of the received data. To take into account the temporal context, the currently received data have been shown from a historical perspective. Results of weather and pollution measurements are displayed on the dashboard as time graphs, which depict data collected during a given time horizon.

### 4.2. Visualization of Temporal Context

While the visualizations of the spatial context and the visual one were relatively well-defined in the framework [37], the visualization of the temporal context strictly depends on the tasks performed by the air station. This is because visualization of the temporal context is usually affected by the features of the monitoring system, such as number of sensors, their importance in terms of performed tasks, measurement methodology and frequency, quick or slow change of measured quantity, and speed of the air station, to mention just a few.

The size of the bottom area limits the number of simultaneously displayed graphs to three. In the case of the Kestrel, data flow coming from sensors can be depicted in the form of time graphs, and WMMS operators can choose which graphs (if any) will be displayed on the dashboard. The time horizon that limits the number of displayed short-term historical data and the interval between time markers depends on the speed of the air station, sensors used, and frequency of measurements. All graphs are labeled with MQTT context to assure unambiguous identification of the source of data depicted in the graphs, even in the case of multiple sensors measuring the same quantity.

### 4.3. Visualization of Dynamics of Change in Spatial Context

The framework [37] offers the functionality of online tracking of a leading quantity. Values of the leading quantity can be associated with the symbol of an air station (inverted triangle) used in the window intended for the positioning service. Then, they are displayed (numerically or drawn as colored circles) in this window on the background of a dynamic OSM map. This functionality allows the monitoring operator to more easily detect problematic situations (such as the appearance of a negative temperature or an abnormally high emission of exhaust gases).

Although the solution [37] offers a framework for the circle markers, this does not solve all the problems encountered in the programming of this functionality due to its strictly task-oriented and hardware-oriented nature. Tasks performed by the monitoring system should be taken into account during all phases of the design and implementation of the circle markers: from the selection of a leading quantity, through the settings of the appearance of the circle markers, to the frequency and time of the markers’ occurrence. Moreover, both the circles’ appearances and the thresholds at which a change in appearance occurs should be adapted to the hardware of the monitoring system, with particular emphasis on the sensor mounted on-board the air station, which performs the measurements of the leading quantity.

In the Kestrel, the concentration of exhaust gases was selected as the leading quantity because of their universality when detecting the traffic-related air pollution. Values of thresholds and the corresponding appearance of the markers have been selected experimentally. Results of this selection are presented in Table 3.

The main criterion for the selection of the appearance and the thresholds was whether changes in layout can be distinguished during fieldwork. The selection was made on the basis of two laptop computers with different screens and smartphones. The selection of the threshold was made taking into account features of the MQ-135 gas sensor and the dynamics of the changes in a cloud of fumes observed during preliminary experiments. As a result, the first threshold value was set to 10 ppm, which is the minimum measuring signal of the MQ-135 gas sensor. Although the maximum measuring signal of the MQ-135 is 200 ppm, we set the last threshold to 105 ppm.

Results of preliminary experiments showed that if measurements are carried out on a steeply rising slope of a cloud of exhaust gases, the MQ-135 sensor reacts too rapidly to make subtle graphical indications useful for a monitoring operator. The setting of the last threshold at 105 ppm means that the fully filled red circle is the indicator of exceeding 105 ppm, and the results of measurements should be observed on the time graph.

## 5. Response Time of the MQ-135 Gas Sensor and its Impact on Flight Plan and Visualization

To measure the concentration of exhaust gases, the Waveshare MQ-135 Gas Sensor board was attached to the SBC as Kestrel’s primary gas sensor. This low-cost electrochemical sensor is able to detect various harmful gases and smoke (Table 1). In order to ensure accurate measurements, the MQ-135 sensor must have good exposure to the surrounding air, which was achieved by hanging the sensor under the air station. The concentration of gases measured by the Waveshare MQ-135 is expressed in parts per million (ppm).

The MQ-135 is an analog sensor (with analog output). The response time of the analog-to-digital converter (ADC) used in the Kestrel for serving this sensor was set to 9 μs. This value was chosen experimentally. We observed that shorter ADC’s response times increased the level of interference. Results of other experiments, focused on the response time of the MQ-135 gas sensor working in Kestrel, showed that in the case of the 9 μs period of probing of the ADC, the gas sensor working on the Kestrel achieved a minimum response time of 3 ms, a maximum response time of 5 ms, and mean one of 4 ms (Table 4).

However, due to the specificity of this sensor, built with the use of a heated ceramic bead impregnated with metal oxide, which works as a variable resistor, the electrical response time given above does not reflect the response time for changes of gas concentration. The MQ-135 sensor’s resistance, working in the presence of gas, stabilizes after less than 1 s, so the response time of the MQ-135 for changes of gas concentration (time to achieving the steady state) is also below 1 s (both the nominal response time and the one measured in the Kestrel system).

Large actual response times mean that the measurements carried out on-the-fly may not fully reflect the reality they have encountered. Since the highly mobile Kestrel’s air station can travel at a relatively high speed, it may happen that measurement on-the-fly starts and ends under completely different conditions. This issue was resolved with the appropriate flight plan, in which the stages of flight were intertwined with stages of hovering. Measurements are conducted continuously, both during flight stages and hovering ones, but only the ones carried out in the hovering stage are considered stable and are believed to be reliable and a faithful representations of the facts. It is worth noting that the same problem with the change of parameters during the flight occurs when the positioning module is used. In the hovering stage, the position correlated with the datum is the best one that the positioning module can calculate at a given moment.

**Figure 3:**
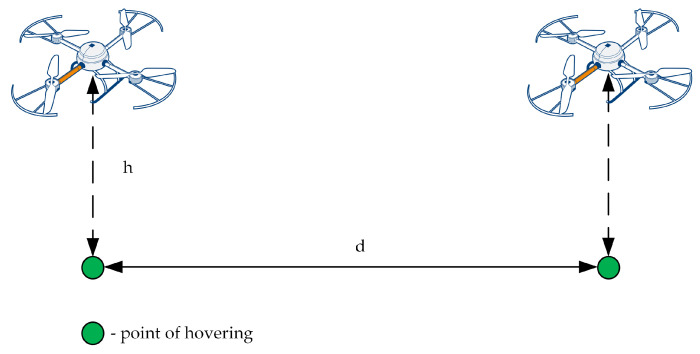
Scheme of flight between two points of hovering.

Each flight begins and ends with a hovering stage, which is understood as being at the measuring point. Figure 3 depicts the air station flying between two stages of hovering. The station moves between the measurement points at a constant height *h* and at a constant speed *v*, depending on the distance between the points and the assumed flight time:

(1) $$v=\frac{dx}{dt}=\frac{d}{t^{f}}$$

where *x* is the displacement, *t* is time, *d* is the horizontal straight-line distance between two consecutive measuring points, and $t^{f}$ is the time of horizontal flight in a straight line between two consecutive measurement points.

In the Kestrel, we have assumed that the distances between measuring points is equal to 5 m. We also assumed that the pair (flight, hovering) last 5 s, i.e., time interval between markers on time graphs. This means that markers always indicate a stable measurement, carried out in the stage of hovering. Moreover, in the case of stable measurements, historical data displayed on the time graph and scaled over time corresponds directly to a hypothetical spatial graph scaled in meters. This facilitates the monitoring operators’ orientation in space and time.

The duration of the pair (flight, hovering) $t^{p}$ is a sum of the flight time $t^{f}$ and hovering time $t^{h}$:

(2) $$t^{p}=t^{f}+t^{h}$$

As the MQ-135 response times are below 1 s, we assume that the hovering stage will last 2 s ($t^{h}$ = 2 s) of each 5 s for a pair (flight, hovering). This allows the Kestrel to have two stable measurements of the concentration of exhaust gases at each measurement point. As a result, the flight time is 3 s. According to (1), the flight speed of the air station moving between measurement points is 1.67 m/s.

The total flight time $T^{f}$ is the sum of all flight times and all hovering times that occur on a given flight route:

(3) $$T^{f}=\sum_{i=1}^{n}t_{i}^{f}+\sum_{i=1}^{n+1}t_{i}^{h}$$

where *i* denotes the *i*-th pair (flight, hovering) and *n* is the number of pairs (flight, hovering) that occur on a given flight route.

Both the flight plan and the visualization of the situational content should be tailored to the needs of the task being performed. As a result, the above customization of the flight plan and the markers on the time graphs should be performed with each change in the conditions of the task.

## 6. Functional Tests

This section presents functional tests of the prototype of the Kestrel, including the description of the patrolled area and flights carried out over this area, with a visualization of bad air quality and good air quality, and a visualization of situational context.

### 6.1. Patrolled Area and Flights

**Figure 4:**
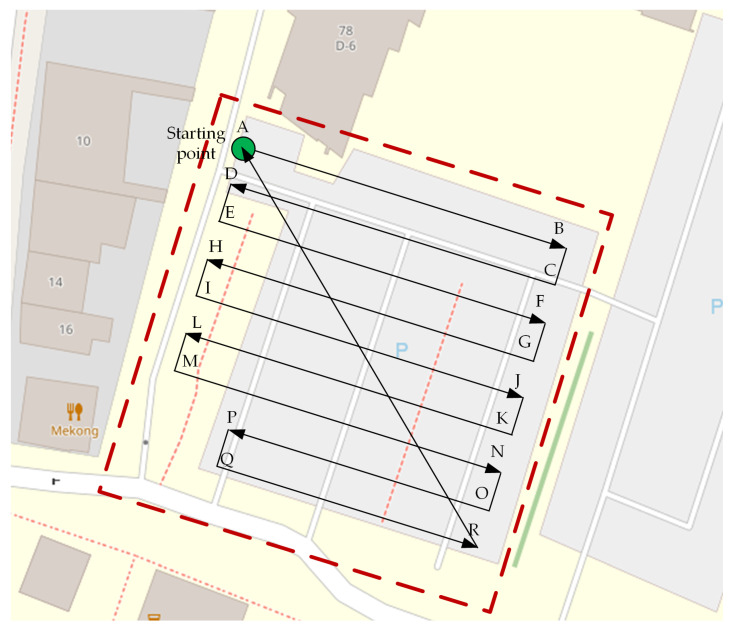
Trajectory of the air station on the map of the parking lot.

As an example of a patrolled area, the parking lot of AGH University of Science and Technology (in Kraków, Poland) was used (Figure 4). Flights in this location are limited by university buildings, external buildings, a row of trees on the east side (the green line on the map), and standing lighting columns in the car park area. All these obstacles were taken into account during the planning of the flight of the air station. An aerial view of the patrolled parking lot is shown in Figure 1b. When this photograph was taken, the station was flying over the lighting columns, at an altitude of 15 m, and the camera was facing the row of trees.

The flight plan considered coverage of the parking area and the need to avoid collisions with any stationary object on the flight trajectory. The air station flew at an altitude of 5 m. To avoid collisions with a tall and branchy tree, growing at the corner of the patrolled area, two sections of the flight route were shortened (O-P and Q-R in Figure 4). The flight plan was prepared using the Mission Planner software tool and, before the air station take-off, it was uploaded into the autopilot software (the ArduPilot open source tool [53,54]).

At the very beginning of each functional test, the ground station was near (between 2 to 5 m) the location described in Figure 4 as the starting point (the point marked with the letter A). The UAV being the IoT carrier is able to carry out completely autonomous missions (including take-off and landing). However, in real, noninsulated environments, there is a high probability of there being various objects in the take-off area. Such objects could interact with the UAV. Thus, for safety reasons, in the take-off part of a flight the manual mode was used. We believe that a human operator would better respond to unpredictable situations than the simple autopilot.

The air station was manually controlled until the minimum flight altitude was reached. Then, the control was switched to automatic mode, in which the ArduPilot controlled the UAV according to the previously prepared flight plan.

The air station took off near the starting point marked with a green circle and, after climbing to an operational height of 5 m (±10 cm of accuracy of the barometric altimeter), flew over the starting point. Then, the Kestrel swept the parking lot according to the trajectory outlined on the map (Figure 4). After achieving the end of the patrolled area (the R point), the air station returned to the starting point.

During experiments, the speed of the air station was set to 1.67 m/s. Every 5 m, the air station hovered for 2 s to make stable measurements. During all flights, every 0.5 s, measurements of the concentration of exhaust gases (expressed in ppm by volume), temperature (in degrees Celsius), relative humidity (in %), and concentration of PM2.5 (in μg/m${}^{3}$) were carried out automatically. Concentration of exhaust gases was put in the spatiotemporal context, which was transmitted to the ground station and visualized there on the dashboard.

### 6.2. Visualization of the Spatiotemporal Context of the MQ-135 Data

**Figure 5:**
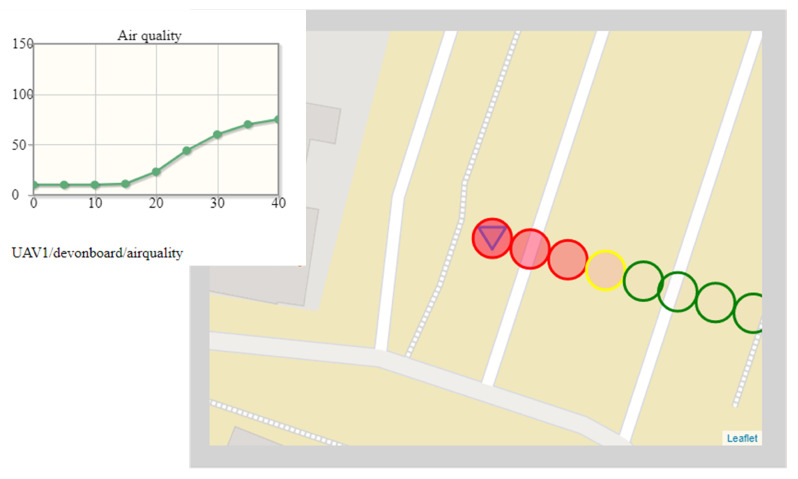
Exemplary situational context (current exhaust gas concentration associated with spatiotemporal metadata and short-term historical data): bad air quality.

Figure 5 presents two fragments of the dashboard (the time graph of the exhaust gas concentration, which partially overlaps on the map of the patrolled place). Both fragments visualize data coming from the MQ-135 gas sensor, their temporal metadata (the fragment on the left), and their spatial metadata (the fragment on the right), and put them all in a common situational context.

The situation depicted in Figure 5 was captured at the turn of May and June, in the morning (about 8 a.m.), when large groups of students had just started their lessons. However, there were still some late persons, and the traffic on the streets to the left and on the top (behind the buildings visible in the map shown in Figure 4) still was busy. The air station moved along the trajectory O-P (the symbol of the air station is at the P point, and the last measurement was carried out at the P point) in a light breeze (2.7 m/s) from the south. The time of day and location of the O-P segment of the flight route in the ventilation corridor, discharging air pollution from the street located behind the left edge of the map showed in Figure 4, suggest that at least the final section of the O-P should lie in an area of bad air quality.

At the very beginning of the O-P section of the flight route, the gas sensor showed almost the minimum concentration of exhaust gases (the first stable measurement made on the O-P section was 10.01 ppm and the third one grew to 10.09 ppm). The first stable measurement plotted on the time graph is 10.1 ppm. The next stable measurements are both visible on the graph (dots) and symbolized on the map (colored circles). The points on the time graphs correspond to the centers of the circles plotted on the map.

Circle indicators changed color successively while the air station was going through areas of different air quality. Green symbolizes both close to the minimum (10.3, 10.4, and 10.5 ppm) and two times larger (20.1 ppm) concentration of exhaust gases, through yellow (44 ppm), to the red that begins with 2% of filling of the circle (60 ppm and 64.9 ppm). The maximum concentration of exhaust gases measured on the segment O-P is 74.9 ppm, and it was also the leftmost circle depicted on the map (the red one with 3% filling).

**Figure 6:**
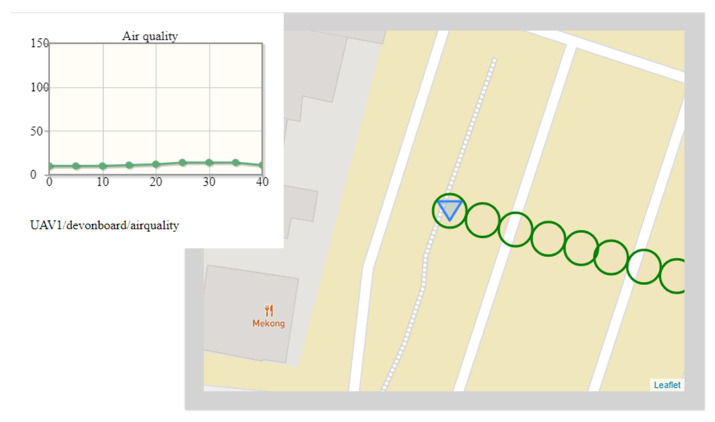
Exemplary situational context (current exhaust gas concentration associated with spatiotemporal metadata and short-term historical data): good air quality.

Figure 6 depicts the situation captured about a week later, in approximately the same weather, at the same place, and at the same time (about 8:30 p.m.). The air station moved along the segment K-L of the flight trajectory (in Figure 5, the location of the air station was close to the point L). A gentle breeze (3.7 m/s) was blowing from the northeast (from the campus of the AGH university), which, in combination with the time of day (no parked cars) and the location of the K-L segment of the flight route (tall buildings separate the K-L from the busy street on the left), caused the entire K-L section of the flight route to be in an area of good air quality.

During the flight of the air station along the K-L line, the stable measurements of concentration of exhaust gases arose from 10.02 ppm, through 10.29 ppm (the last value outside a graph), 3 ppm (the first value shown on the graph), to the maximum value of 15.1 ppm. Then, concentration of exhaust gases falls, and the last presented value was 12 ppm (the last dot on the time graph and the leftmost green circle on the map). The next value of exhaust gas concentration was 11.9 ppm (stable measurement at the point L, not depicted in Figure 6).

### 6.3. Visualization of the Wide Context of the MQ-135 Data

Figure 5 and Figure 6 put the data coming from the MQ-135 gas sensor in a spatiotemporal context. However, data coming from the other sensors of the Kestrel (Table 1), and visual information captured from the 4K camera enable visualization of the current concentration of exhaust gases in a wider context. Such wider context is displayed on the dashboard as mandatory visual information about the surroundings and optional time graphs of data from sensors. The same MQ-135 sensor data as in Figure 5 and Figure 6 are presented in wide context (spatial, temporal, weather, and visual) in the figures below.

**Figure 7:**
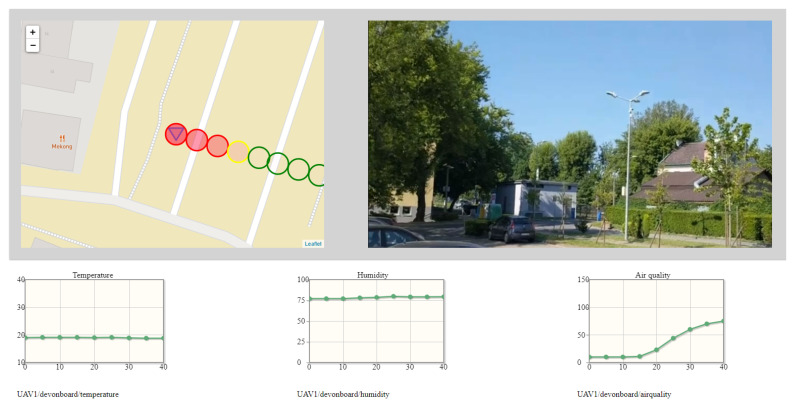
The dashboard, fragments of which were used to prepare Figure 5.

**Figure 8:**
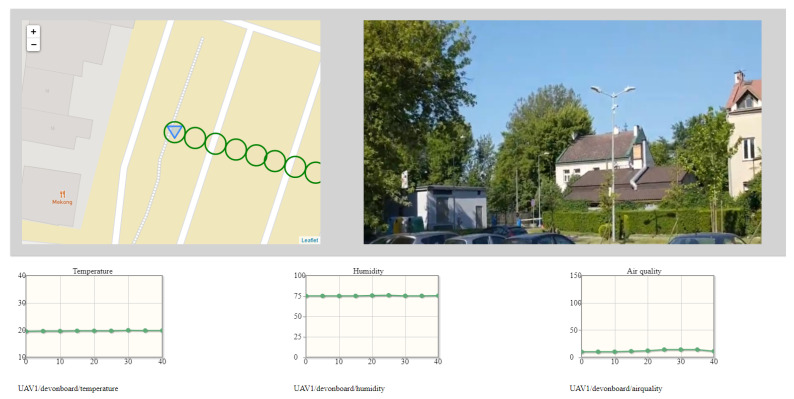
The dashboard, fragments of which were used to prepare Figure 6.

Figure 7 and Figure 8 depicts the wide situational context captured during patrol flights. The gimballed 4K camera was directed to showing a neutral footage. However, the image detail is enough to conclude that the monitoring operator should be able to locate potential sources of excessive emission of exhaust fumes or to detect security concerns. Symbols on maps are placed to avoid overlapping of air quality indicators. The exemplary set of graphs includes measurements of temperature, humidity, and air quality. As air quality, exhaust gas concentrations expressed in ppm is understood.

Both the graphs and the pace of the drawing of symbols on the map are customized to enable a good presentation of a context. Graphs are replotted every 0.5 s, according to new data coming from the air station. The dot on the graph is placed every 5 s and indicates the last, most-stable measurement of a hovering stage. The time horizon of the graphs was set to 40 s. The speed of the air station (1.7 m/s), with the interleaving of flight stages and hovering ones, and the measurement frequency were decisive for this setting. In practice, the air station fled with the speed of from 1.6 to 1.7 m/s, depending on the wind force and speed, which is not noticeable in the graphs.

The time graphs have an auxiliary function in the process of detection of the excessive emission of exhaust gases, except the time graph of the concentration of exhaust gases, which is especially useful when the concentration exceeds 105 ppm (the value indicated by 100% filled red circle). Nonetheless, they are able to provide detailed knowledge about the environment. As an example, Figure 7 shows a slight drop in temperature and a slight increase in humidity as the air station enters the shaded area. Increase in the PM2.5 (not depicted in Figure 7) suggests fumes of a Diesel engine.

Time graphs, offered by the Kestrel, are optional, and monitoring operators have to choose which ones they want to watch and whether they want to watch any at all. However, the choice must take into account the completeness of the information that will be displayed on the dashboard and must allow the monitoring operator to observe a complete picture of the situation.

To avoid overloading the dashboard with self-evident information, especially when the dashboard will be displayed on small devices (such as a smartphone), graphs presented on the dashboard have no units of measurement. A short, simple “dry run” test conducted by one of the authors show that the ordinates are intuitively understandable. After a short briefing, the abscissae also become understandable.

The time resolution of measurements is a half second (two measurements of each environmental parameter per second). This resolution is quite enough for the “hunt” application if the Kestrel’s air station is moving at the assumed speed (1.67 m/s) and the hovering stages last the assumed time of hovering (2 s)—at least in the case of the patrolled parking lot, because the air station speed and the time of hovering is (and should be) adjusted to the patrolled area. The full cycle of the flight over the patrolled area (from point A to point R and then back to point A) takes above 740 s (about twelve and a half minutes), including hovering stages. In the case of larger patrolled areas this may be insufficient and the speed of the air station must be then increased. This will entail the need to increase the sampling frequency (and, as a result, decrease the sampling time) and to shorten the time of hovering.

In our monitoring system, the sensors’ sampling frequency was set to 25 Hz; then, it was reduced to 2 Hz during the polling of sensors by the SBC so that there was enough margin to increase the time resolution of measurements. However, the two-second hovering time was set to double the approximate actual response time of the MQ-135 gas sensor in the Kestrel monitoring system (up to 1 s—see Table 4 for details), in order to achieve accurate and complete information (due to measurements carried out in a stable environment). Thus, shortening this time, especially under the 1 s, which is the gas sensor’s actual response time, should be well thought out.

**Figure 9:**
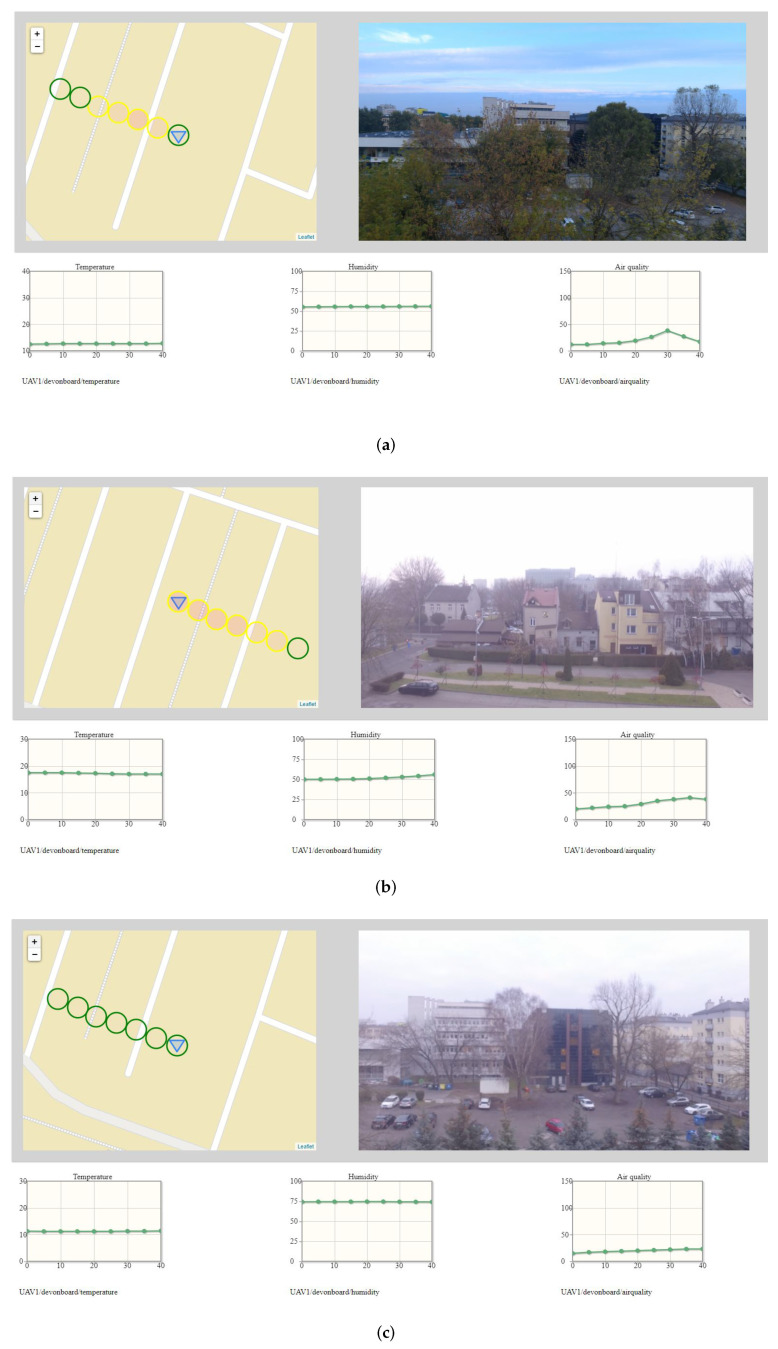
Typical situational contexts observed during Kestrel’s flights: (**a**) instantaneous worsening of air quality; (**b**) from good to worse air quality; (**c**) continuously good air quality.

Due to the limited time that cars that do not meet the exhaust purity standards are driving around the city, the situation presented in the Figure 5 and Figure 7 is rare. The situational context observed during the patrol flights typically boils down to detecting the levels of the concentration of exhaust gases marked on the map with green circles, and in the worst case with yellow circles. Figure 9 shows three typical situational contexts captured several weeks apart (beginning of September, late September, November) during flights at an altitude of 15 m.

In the situation depicted in Figure 9a, the air station passed through an area of moderate air quality separating two areas of good air quality. The area of moderate air quality could be, for example, a plume of fumes from a single car’s exhaust pipe. The maximum detected concentration of exhaust gases was between 35 ppm and 45 ppm. The shape of the graph of exhaust gas concentration suggests that the fixed measurement point was above the area of largest concentration of exhaust fumes.

In Figure 9b, the air station leaves an area with low exhaust gas concentration (campus of the AGH University) and approaches a street with moderate air quality. Air quality in the vicinity of the street improves slightly as it is naturally well-ventilated. In this case, the maximum detected concentration of exhaust gases also was between 35 ppm and 45 ppm.

Figure 9c presents a situation often observed during evaluation flights, when the air station continuously flew through an area with good air quality. As signaled by empty green circles drawn on the map, the detected concentration of exhaust gases always was below 15 ppm.

## 7. Retrospective Analyses

Data coming from the air station can be stored by the ground part of the Kestrel (locally or in a cloud) and then be used for retrospective analysis. We suggest that the retrospective analyses should be made mainly on the basis of the results of stable measurements of the concentration of exhaust gases collected at fixed measurement points (last stable measurements collected at stages of hovering).

While the previous section was devoted to the online detection of the excessive emission of exhaust gases, this section is focused on off-line detection. In the following subsections, results of five field experiments are presented and then discussed in terms of excessive emission, positive detection and false positive ones, and normal operation of the patrolled parking lot.

### 7.1. Experiments and Results

To evaluate the possibility of offline detection of the excessive emission of exhaust gases, measurements of the concentration of exhaust gases collected at the fixed measurement points during five experiments—Experiment 1 to Experiment 5—were carried out, in which the air station swept the parking lot flying at an altitude of 15 m. As in the previous section, the patrolled area was the parking lot of the AGH University of Science and Technology (situated in Kraków, Poland). Each experiment was carried out on a separate day. The aims of the experiments and the achieved side effects are summarized in Table 5. This table also shows the mean wind speed and direction, measured by a weather station located about 100 m from the north edge of the parking lot.

**Figure 10:**
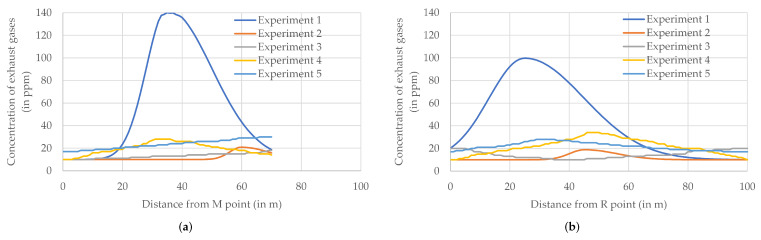
Concentration of exhaust gases (in ppm) measured at fixed points in selected sections of the flight route: (**a**) M-N; (**b**) R-A.

Figure 10 summarizes the instantaneous values of the concentration of fumes, measured by the air station in sections M-N and R-A of the flight route (Figure 4), presented as functions of the horizontal distance from the starting point of a given section (point M and point R, respectively). The section M-N is the first long section crossing the parking lot from the south, located about 20 m from the edge of the parking lot and the local distributor road, on the extension of the local ventilation corridor (the gap between the compact street development) mentioned in the previous sections. The return section R-A passes the parking lot diagonally and crosses other sections of the flight route, including the M-N section.

Statistical properties of the exhaust gas concentration (minimum, maximum, arithmetic mean, median) are summarized in Table 6. Statistics included in this table were calculated for the M-N section and the R-A section.

### 7.2. Excessive Emission

Experiment 1 was devoted to the detection of an excessive emission of exhaust gases. The take-off of the air station was correlated with a one-off excessive emission of exhaust gases near the M-N section of the flight route. The source of this emission was the starting of a cold Diesel engine of a classic car. As shown in Figure 10, during the retrospective analysis, the existence of the excessive emission is easily detectable in the background of emissions typical for the urban environment (experiments 3 and 4), even if the background level of the fume concentration is heightened (experiment 5). Excessive emissions also can be detected through statistical analysis (Table 6), both as an abnormally large peak value (several times larger than usually measured) and as an abnormal raising of the arithmetic mean and the median (a few times larger than usual) averaged over a given section of the flight route.

**Figure 11:**
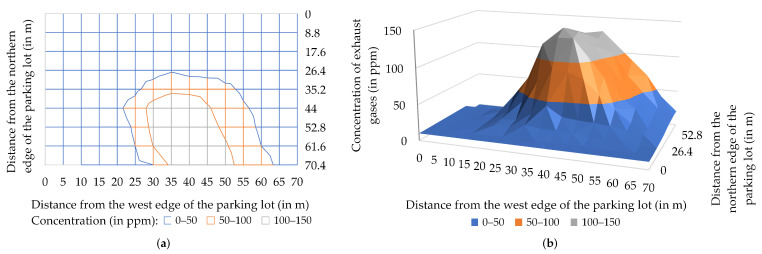
Concentration of exhaust gases (in ppm) measured during experiment 1: (**a**) wireframe contour chart; (**b**) three-dimensional surface chart. For a better presentation, the surface chart (**b**) is a mirror image of the original chart (in the front there is the northern edge of the parking lot—in the rear, the southern edge; on the left, the west edge; and on the right, the eastern edge).

It is worth remarking that the finite speed of the air station gave enough time for the cloud of fumes to spread around the area before the station was able to reach the point of emission. Moreover, the cloud of fumes is shaped by wind. As a result, this point can only be located with a certain approximation, as an area of highest concentrations of exhaust gases, and the analysis should also take into account wind speed and direction. In experiment 1, a moderate breeze (7 m/s) blowing from the northwest pushes a cloud of exhaust gases towards the edge of the parking lot (Figure 11). The maximum of the concentration graph drawn for the M-N section of the flight route is located approximately in the middle of this section (Figure 10), which indicates that the source of the emission should be searched for in this area. This conclusion is consistent with the ongoing observation—the classic car was parked more or less in the middle of the parking lot, close to the M-N line.

### 7.3. Lack of Long-Term Effects of a Single Excessive Emission

Experiment 2 was designed for checking whether the one-off excessive emission is able to affect long-time measurements. In this experiment, the take-off of the Kestrel’s air station was delayed by about half an hour compared to the triggering of the one-off emission. The time interval between triggering by the emission source and the air station’s take-off corresponds to two parking patrolling cycles (one cycle takes about twelve and a half minutes). The emission source was located in the A-B section, the farthest from the ventilation corridor, in a quiet place between the buildings, which guaranteed slow spreading of fumes in the given weather condition (clear weather, a light breeze blowing from the south). The place and time of the experiment minimized the probability of other emissions.

As depicted in Figure 10, there is no sign of excessive emission in the measurements of the concentration of exhaust gases carried out during this experiment. Results obtained in the M-N section show that the air station flying along the M-N section registered the minimum concentration of exhaust gases in the hovering stages (as a reminder, the minimum reading of the MQ-135 gas sensor is 10 ppm). Results obtained in section R-A show that the air station crossing the parking lot diagonally registered the minimum concentration of exhaust gases when approaching point A. This was confirmed by statistical results listed in Table 6. The medians of the results of measurements carried out at fixed points both in the M-N segment and in the R-A segment was equal 10 ppm.

In summary, the single one-off large source of emission was not able to affect the Kestrel’s detection abilities in the second patrolling cycle after the emission (at least in the case of the parameters used by the experiment, e.g., the speed of the air station and the length of the patrol route). This reduces the likelihood of false positives and increases the risk of false negatives.

### 7.4. Positive Detection and False Positive Detection

During experiment 2, two heightened concentrations of exhaust gases were registered and then depicted in Figure 10. The first one was located in the M-N section (near the N point) and the second one in the R-A section (in the middle of the parking lot). The statistics of the concentration of exhaust gases (Table 6) show a small increase in the mean (up to 11.7 ppm) in both analyzed segments of the flight route, and small maximum values (low-level peaks, marked with green circles on the map). Such a small increase is likely to go unnoticed or ignored by the monitoring operator, but during retrospective analyses, it allows one to hypothesize that two sources of weak emissions were triggered during the flight of the Kestrel’s air station or shortly before the flight.

For most of the flight of the Kestrel air station along the M-N section, the concentration recorded in the hovering stages was equal to the MQ-135 sensor’s minimum (10 pmm). This changed when the air station left the point of measurement at 50 m from the M point and at the next point of measurement (55 m from M point, 15 m from N point), where an increased concentration of exhaust gases was registered. At 60 m from the M point (10 m from N point), the exhaust gas concentration achieved a maximum (21 ppm). Measurements carried out at the N point (the fixed point of measurements at the end of M-N section) showed 15 ppm.

**Figure 12:**
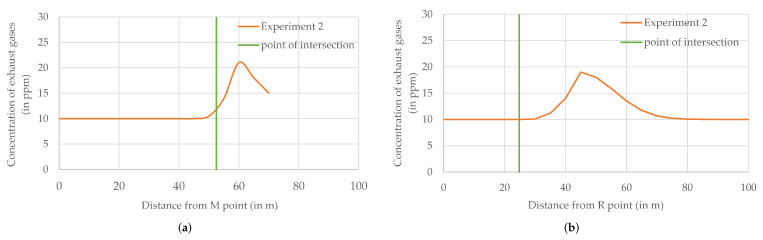
Concentration of exhaust gases (in ppm) during experiment 2 in (**a**) M-N section of flight route; (**b**) R-A section of flight route.

Exactly in the middle between the 50th and 55th meter of the M-N section is the point of intersection of the M-N trajectory with the R-A one (Figure 12). Measurements made in the flight stage by the air station following the M-N trajectory showed 11.8 ppm at this point. However, the air station passing the intersection point 3 min later registered at that location the minimum value of the exhaust gases concentration (10 ppm), presumably due to the southerly wind blowing away the fumes. The largest concentration of fumes in the R-A section (19 ppm) was measured in the hovering stage at 45 m from the R point (55 m from the A point, and 20 m from the point of intersection). This is an important argument supporting the hypothesis of the detecting of two separate sources of exhaust gases.

The hypothesis of detecting two sources of pollution was not confirmed by visual observation. During experiment 2, only one source of fumes, i.e., the one near the N point, was active. It was a car parked between K-L and M-N lines, at the left side of an inner road (see Figure 4). As a result, the source of pollution detected in the R-A section remains unknown. It could have been a car that left the parking lot unnoticed by the monitoring operator when the air station was prepared for a flight. However, most probably it was a false positive detection, caused by, as an example, local air turbulence that blew a cloud of exhaust gases over twenty meters further.

### 7.5. Normal Operation of the Patrolled Parking Lot

Experiments 3, 4, and 5 were chosen from a series of evaluation flights. Selection was made in terms of presentation of the typical situations that occurred during normal operation of the patrolled parking lot.

Usually, cars are parked in places that allow drivers and passengers to have a shorter path to their destinations. Thus, the heightened concentration of exhaust gases is observed in locations close to University’s buildings (experiment 3), i.e., on the north side of the patrolled area (A point) and on the east one (R point and N point). If these best parking spaces are occupied, drivers park their cars in the center of the parking lot (experiment 4). Statistics of the concentration of exhaust gases (Table 6) show a small concentration during experiment 3 (means and medians not exceed 15 ppm, peaks up to 20 ppm), meaning that only green circles appeared on the map. During experiment 4, the concentration of fumes was larger (means and medians up to 22 ppm, peaks up to 34 ppm), and when the air station was in the middle of the parking lot, the markers on the map turned yellow.

Krakow is a large city (the second largest city in Poland) situated in a valley between hills on a riverbank, so smog is not an unusual phenomenon, especially in autumn. As a result, the sometimes elevated background level of the concentration of fumes was observed. Experiment 5 shows a situation where the background concentration of exhaust gases was elevated to 17 ppm (Table 6). This level of fumes concentration was observed at almost all edges of the parking lot, except the last one, where the impact of weak sources of pollution was observed in the M-N section, close to the N point, and in the middle of R-A section. Peak values did not exceed 30 ppm (Table 6), which suggests that the heightened concentration of fumes was the compound effect of elevated background level and a weak source of emission.

## 8. Discussion

Table 7 compares the Kestrel with other IoT systems for pollution monitoring, known from the literature. Most of these systems measure both gaseous pollutants and particulate ones, and the Kestrel follows this trend. These systems use different low-cost gas sensors, but the Waveshare MQ family [12,20,23,24] and the Bosch BME family [11,21,24] dominate. The Kestrel uses the Waveshare MQ-135 gas sensor and the HM3301 sensor for measurements of particulate matter, while other solutions use the Dylos DC1700 one [25,26] and the low-cost Alphasense OPC family [21,27]. In general, in terms of used sensors, the Kestrel is similar to other solutions listed in Table 7. However, unlike these solutions, it also uses the UAV’s camera to improve situational awareness of the monitoring operator.

The biggest difference is observed in the online visualization of incoming data. In systems listed in Table 7, user interfaces (UI) were usually built as Web pages [11,18,19,20,24,25,26,27] or mobile applications [12,20,21]. In a few cases, integrated displays were used [24]. Some of the solutions do not use UIs, and data coming from these systems could be only visualized offline [21]. The Kestrel uses a web page as the user interface; however, the visualization delivered by this system is context-oriented. Thus, although almost all of the mobile IoTs listed in Table 7 are equipped with a positioning system, the Kestrel is the only one that marks the place of measurement on the map and shows a graphical indicator of the current air quality (concentration of exhaust gases). The Kestrel is also the only one that shows video data associated with measurements coming from sensors.

One of the common features of pollution monitoring systems is various types of time graphs of measured quantities. They are from one-dimensional graphs, which show only the time of measurement [12], through the most common two-dimensional ones [11,20,24,25,26], to three-dimensional spatial maps (e.g., AQI map at different time stamps [18,19]). However, these visualizations are focused on making a live view of data from sensors visible on the graph and not on showing the situational context. The only exception was the system designed for real-time monitoring of multiple air pollutants, which presents a kind of information fusion that integrates the UAV’s geo-location data, time data, and sensor data [27]. Geo-location data are presented on the background of the photography of the monitored area. The same kind of fusion, but extended with video data, is the core of online visualization carried out on the Kestrel’s dashboard.

## 9. Conclusions

The Kestrel is a flying IoT system able to monitor air pollution and current weather conditions. The system is built on the UAV and WebRTC-based platform, which, on the one hand, assures high mobility and the possibility to conduct automatic missions, and, on the other hand, offers an easy to update, portable Web application in the air station and a modern, developmental method of communication. The platform was retrofitted with a set of sensors, including the MQ-135 measuring the concentration of exhaust gases, which required updating the software of the sensor service of the platform.

The important part of the Kestrel is the WebRTC Multimedia and Monitoring Station, which performs online visualization of data coming from the sensors and puts them together with their spatial, temporal, and visual metadata in a common situational context. This context is displayed on the dashboard, which is customized to Kestrel’s monitoring task. Customizing to the task also applies to the mission plan, e.g., by including in the plan of the flight mission the stages of flight and hovering over measurement points. The latter allows the MQ-135 sensor to have steady state measurements.

To evaluate our system, field experiments were carried out on an open parking lot. The online monitoring was evaluated basing on a series of experiments. Two of them presented extreme cases: bad air quality (in practice: the transition from good air quality through medium air quality to bad air quality) and continuously good air quality. Three other experiments showed typical situations (moderate and good air quality). The results of the air quality measurements, understood as measurements of the concentration of exhaust gases, were shown both in the narrow (spatiotemporal) situational context and the wide one (spatiotemporal, visual, and weather one). These results are supplemented with retrospective analyses carried out on the basis of five other experiments that presented excessive emission of exhaust gases, false positive detection, and normal operation of the patrolled parking lot. The above results show that the Kestrel offers good situational awareness; so, the system operator should be able to easily detect excessive emissions of exhaust gases.

The proposed system has the potential to fill the gap between professional systems of environmental monitoring and systems based on voluntarily carried devices or crowdsensing. Future investigations will focus on the use of data coming from the Kestrel for the retrospective analysis of air pollution in the patrolled area.

## Figures and Tables

**Table 1:** Input devices used in the air station of the Kestrel system.

Device	Type	Comments
**Kestrel-specific devices**		
gas sensor	Waveshare MQ-135 (Waveshare Electronics, Shenzhen, China)	NH${}_{3}$, NO${}_{x}$, alcohol, benzene, smoke, CO${}_{2}$, and other gases
temperature and humidity sensor	Measurement HTU21D (Measurement Specialties, Hampton, VA, USA)	fast response sensor
PM2.5	Seeed Studio HM3301 (Seeed Technology Co. Ltd., Shenzhen, China)	laser sensor, particle size: 2.5 μm, 5 μm, 10 μm
**framework devices**		
4K camera	Manta MM9359FS (Manta S.A., Warsaw, Poland)	live streaming via USB
positioning module	Waveshare SIM7000E (Waveshare Electronics, Shenzhen, China)	NB-IoT LTE GPS HAT

**Table 2:** Data and metadata transmitted between the air station and the ground one.

Data	Device	Served by	MQTT Topic
concentration of gases	Waveshare MQ-135	sensor service	UAV1/devonboard/airquality
current position	Waveshare SIM7000E	positioning service	UAV1/devonboard/position
current position	buffer	sensor service	as the accompanied data
humidity	Measurement HTU21D	sensor service	UAV1/devonboard/humidity
temperature	Measurement HTU21D	sensor service	UAV1/devonboard/temperature
time	system clock	sensor service	as the accompanied data
time	video card clock	WebRTC video service	not applicable
PM2.5	Seeed Studio HM3301	sensor service	UAV1/devonboard/PM
video frame	Manta MM9359FS	WebRTC video service	not applicable

**Table 3:** Circle appearance according to the concentration of exhaust gases (in ppm).

Concentration of Exhaust Gases	Border Color	Percentage Filling of the Circle
10 (minimum)	green	0%
15	green	1%
25	yellow	5%
35	yellow	10%
45	red	20%
55	red	30%
65	red	40%
75	red	50%
85	red	60%
95	red	80%
105 and more	red	100%

**Table 4:** Response times of the MQ-135 gas sensor.

Response Time	Measured in Kestrel			Nominal
	Minimum	Maximum	Mean	
Electrical	3 ms	5 ms	4 ms	-
Actual	0.87 s	0.95 s	0.92 s	≤1 s

**Table 5:** Properties of experiments.

Experiment	General Description	Side Effect	Wind	
			Speed	Direction
1	Excessive emission		7 m/s	northwest
2	Delayed measurements	False detection	2.6 m/s	south
3	Normal operation		3 m/s	northeast
4	Normal operation		3.8 m/s	northeast
5	Normal operation	Heightened level ^1^	7 m/s	southwest

^1^ Heightened level of background concentration of exhaust gases.

**Table 6:** Concentration of exhaust gases (in ppm) during a given experiment, in sections M-N and R-A.

Experiment	Section M-N				Section R-A			
	Min	Max	Mean	Median	Min	Max	Mean	Median
1	10	139	58.5 ± 11	44	10	99	44.4 ± 6.3	32
2	10	21	11.7 ± 0.8	10	10	19	11.6 ± 0.5	10
3	10	18	12.6 ± 0.5	12	10	20	14.3 ± 0.6	13
4	10	28	19.1 ± 1.2	18	10	34	21.5 ± 1.3	22
5	17	30	22.7 ± 0.9	22	17	28	21.5 ± 0.6	21

**Table 7:** Comparison of the Kestrel with related work.

Paper	Pollutants	Sensors	Positioning System	Online Visualization			
				Video	On a Map		Time Graphs
					Location	Quantity	
[11]	IAQ, CO${}_{2}$, TVOC	BME688, SGP30	no	no	no	none	yes
[12]	fire smoke, CO${}_{2}$, ammoniac, benzene, alcohol	MQ-135	no	no	no	none	no
[24]	H${}_{2}$	MQ-8, BME680	no	no	no	none	yes
[25,26]	PM2.5	DC1700	GPS	no	no	none	yes
[18,19]	CO, NO, SO${}_{2}$, O${}_{3}$, PM2.5, PM10	unknown	GPS	no	no	none	no
[20]	VOC	MQ-135	no	no	no	none	yes
[21]	AQI, PM2.5	BME680, OPC-N3	GPS	no	no	none	no
[13]	CO, CO${}_{2}$, NO, NO${}_{2}$, O${}_{2}$, O${}_{3}$	N.A.	GPS	no	no	none	no
[27]	PM1, PM2.5, PM10	OPC-N2, NO2-B43F	GPS, GLONASS, BeiDou	no	no	none	yes
[14]	O${}_{2}$, CO${}_{2}$	LuminOx, K30 SE-0018	GPS	no	no	none	no
This Paper	NH${}_{3}$, NO${}_{x}$, alcohol, benzene, smoke, CO${}_{2}$, PM2.5	MQ-135, HM3301	GPS, GLONASS, BeiDou	yes	yes	concentration of exhaust gases	yes

## Data Availability

Not applicable.

## Abbreviations

The following abbreviations are used in this manuscript:

ADC	Analog-to-Digital Converter
AQG	Air Quality Guidelines
CCC	Command and Control Console
I${}^{2}$C	Inter-Integrated Circuit
IoT	Internet of Things
MQTT	Message Queue Telemetry Transport
OSI	Open Systems Interconnection
OSM	OpenStreetMap
PM	particulate matter
ppm	parts per million
UAV	Unmanned Aerial Vehicles
UART	Universal Asynchronous Receiver/Transmitter
UTF-8	8-bit Unicode Transformation Format
WebRTC	Web Real-Time Communications
WHO	World Health Organization
WLAN	Wireless Local Area Network
WMMS	WebRTC Multimedia and Monitoring Station
